# Supplementary figures and images for: Disrupting cortico-cerebellar communication impairs dexterity
Source: eLife. 2021 Jul 29;10:e65906. doi: 10.7554/eLife.65906 (PMC8321550; doi:10.7554/eLife.65906)

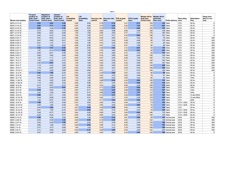

Supplement: Supplementary file 1. [file elife-65906-supp1.numbers.zip › supplementary_file_1.numbers/preview-web.jpg]

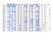

Supplement: Supplementary file 1. [file elife-65906-supp1.numbers.zip › supplementary_file_1.numbers/preview-micro.jpg]

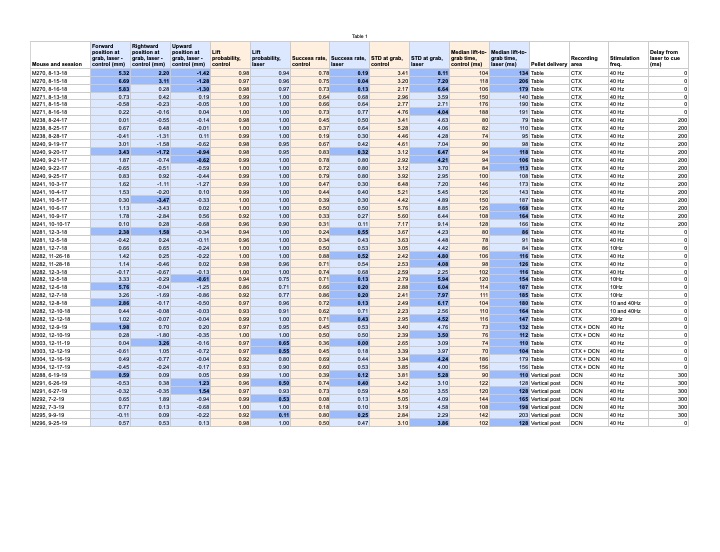

Supplement: Supplementary file 1. [file elife-65906-supp1.numbers.zip › supplementary_file_1.numbers/preview.jpg]
